# Open-source tubing-free impeller pump platform for controlled recirculating fluid flow for microfluidics and organs-on-chip

**DOI:** 10.1016/j.ohx.2025.e00673

**Published:** 2025-07-04

**Authors:** Sophie R. Cook, Erin E. Lawrence, Parastoo Sakinejad, Rebecca R. Pompano

**Affiliations:** a Department of Chemistry, University of VA (UVA), VA, USA; b Department of Chemical Engineering, University of VA (UVA), VA, USA; c Department of Biomedical Engineering, University of VA (UVA), VA, USA

**Keywords:** Multi-organ-on-chip, 3D printing, Bioreactor, Stir bar, Micropump

## Abstract

Fluid flow is utilized in many microscale technologies, including microfluidic chemical reactors, diagnostics, and organs-on-chip (OOCs). In particular, OOCs may rely on fluid flow for nutrient delivery, cellular communication, and application of shear stress. In order for microscale flow systems to be readily adopted by non-experts, a tubing-free, user-friendly pump would be useful, particularly one that is simple to use, affordable, and compatible with cell culture incubators. To address these needs, here we share the design and fabrication of an impeller pump platform that provides recirculating fluid flow through a microfluidic loop without the need for tubing connections. Flow is driven by rotating a magnetic stir bar or 3D-printed impeller in a pump well, using magnets mounted on a DC motor. The DC motors used produce negligible heat output in a compact system, making it compatible with cell culture incubators. The pump platform accommodates user-defined microfluidic or OOC device geometries, which may be easily customized by 3D printing. Furthermore, the system is easily assembled from low-cost materials and simple circuitry by someone with no prior training. We demonstrate the ability of the platform to drive recirculating fluid flow in a microfluidic device at well-characterized flow velocities ranging from µm/s to mm/s for use with microfluidic technologies. Though designed with OOCs in mind, we envision that this platform will enable users from ranging disciplines to incorporate fluid flow in customized microscale technologies.

## Graphical abstract

**Figure:**
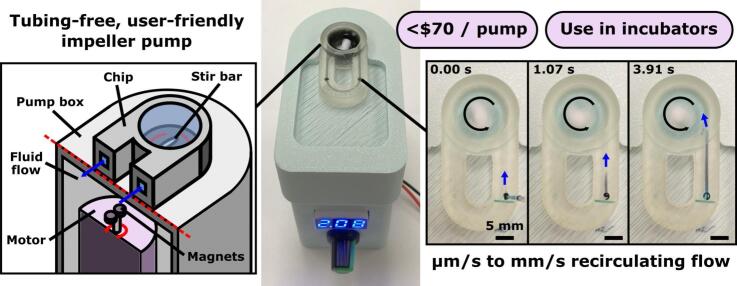

**Specifications table**.

**Table:** 

Hardware name	Microfluidic impeller pump
Subject area	• Engineering and material science • Chemistry and biochemistry • Biological sciences (e.g., microbiology and biochemistry)
Hardware type	• Biological sample handling and preparation • Handling and manipulation of fluids
Closest commercial analog	No commercial analog is available.
Open source license	CC BY-NC 4.0
Cost of hardware	*$68 USD*
Source file repository	https://doi.org/10.17632/5njjj7kpys.1

## 1 Hardware in context

Recirculating fluid flow is a key component in many types of microscale reactors, with applications ranging from flow chemistry to organs-on-chip (OOC). In each case, control over flow rate is needed, ideally without adding significant complexity to the experimental system. Here we focus on the need for flow through OOC, in which flow may be applied to recapitulate the physiology and pathology found *in vivo* and to connect multiple organs for delivery of secreted signals from one tissue to another [[1], [2], [3]]. Over the last 10+ years, the methods for fluidic control in single- and multi-OOCs have improved. However, many of the commercially available pump methods, like syringe or peristaltic pumps, are challenging for non-experts like researchers in a biology-focused lab to use due to extensive tubing connections, large overall size, and high cost. For example, prior work in our lab utilized bulky commercially available peristaltic pumps to generate linear or recirculating fluid flow in organ-on-chip devices [4,5]. Although they provided excellent control over flow rates, the expensive pumps were incompatible with cell culture incubators due to heat production, and they required many long external tubing connections, which increased experimental preparation time and failure rate due to bubbles, contamination, or leaky connections. Many research labs have developed novel pumps to address these issues [[6], [7], [8], [9], [10], [11], [12], [13]]. However, there is an unmet need for customizable pumps that can be adapted for different applications and are low-cost, are easy to use with limited tubing, and have a small footprint.

Here, we describe how to build and characterize a tubing-free and user-friendly microscale impeller pump platform, to drive recirculating fluid flow through a companion microfluidic chip at well-characterized flow velocities ranging from µm/s to mm/s. Here we define tubing-free as not requiring external tubing, though we acknowledge that the microfluidic channels in the companion chip may be considered to be tubing in some fields. The absence of external tubing eliminates common problems of leaky connections and reduces the risk of contamination and bubble formation. To use, one need only to fill a chip with liquid, add a stir bar, and place it on top of the pump housing, which is plugged in to a wall outlet. A turn of a knob sets the pump speed. Results from prior iterations of this system were described in recent publications [14,15], and here we share detailed protocols for fabrication to make this technology broadly available for other researchers. Major design goals for the microfluidic impeller pump included 1) a user-friendly interface with no external tubing, limited wires, and simple controls; 2) controllable recirculating fluid flow; 3) customizable device geometry to accommodate a wide range of different applications; 4) low overall cost; 5) small pump footprint; and 6) compatibility with cell culture incubators due to low heat output.

## 2 Hardware description

### 2.1 Working principle of impeller pump and design of motor assembly

The microfluidic impeller pump takes inspiration from centrifugal water pumps used in the fishing industry, artificial heart pumps, and the on-chip kinetic pump for organs-on-chip applications, where the pump systems drive fluid flow by centrifugal force [6,7,16,17]. Here, the pump drives recirculating fluid flow on-chip by rotating a magnetic impeller, often a simple stir bar, within a fluid-filled pump well that is connected to a loop of channels. As the impeller rotates, it forms a vortex in the fluid, an effect commonly observed when mixing liquid in a beaker with a magnetic stir bar. The vortex pushes liquid into and pulls it from the connected loop of channels, resulting in recirculating flow in one direction within the device. The range of channel velocities achievable with a specific device is dependent on the device geometry, impeller geometry, photoresin used for device and/or impeller, and media viscosity. Once these features are set, the pump provides a range of channel speeds as a function of the rotation speed of the impeller (rotations per minute, RPM).

**Fig. 1:**
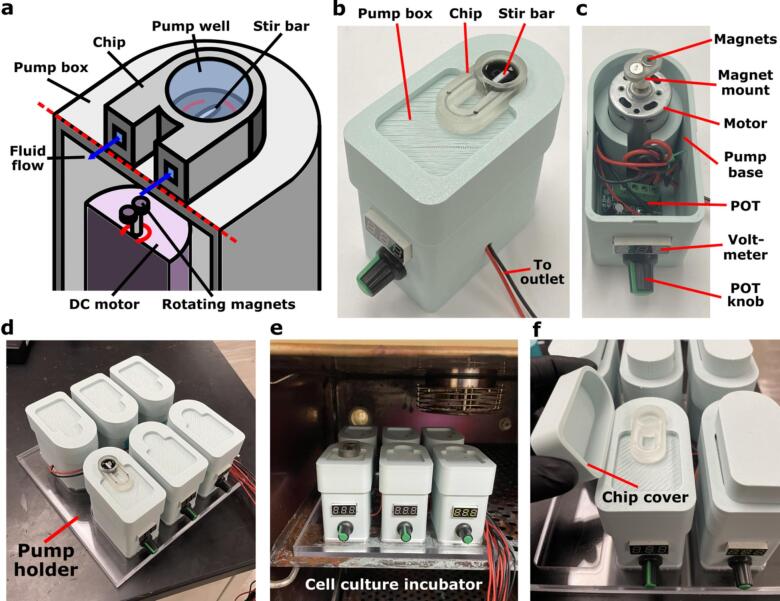
**Overview of the tubing-free impeller pump platform.** (a) The impeller pump drives recirculating fluid flow on-chip by rotating a magnetic stir bar within a cylindrical pump well within the device. (b) An image of a device loaded on to the impeller pump. The chip was filled with food dye and a 10 mm stir bar was added to the pump well. (c) An image of the interior of the pump platform, showing the motor, mounted magnets, voltmeter, and POT within the 3D-printed base. (d,e) An image of six impeller pumps resting on a laser-cut acrylic pump holder on the bench (d) and inside a standard cell culture incubator (e). (f) An image of the chip cover being added to an impeller pump to limit evaporation.

Similar to a conventional stir-plate, the impeller is driven using a rotating magnetic field, thus avoiding the need to insert a rotor into the microdevice. While a suitably miniature stir plate could be used to drive the impeller, here we designed a bespoke pump platform and housing that provides rotating magnets at a well-controlled distance from the impeller (Fig. 1a-c), a built-in positioning system to align the impeller over the magnetics (Fig. 1b), and a small footprint. The platform was designed to provide reasonable water-proofing and low heat emission for use in cell incubators (see section 4.1) [15]. The rotation of the impeller is controlled by rotating magnets mounted on a DC motor that is controlled using a potentiometer (POT) and voltmeter for voltage control and readout, respectively. The motor and electronic components of the circuit are organized within a plastic housing, which is fabricated by fused deposition modeling (FDM) 3D printing (Fig. 1c). The entire circuit is powered using a power connector and adapter that plugs into a standard wall outlet.

When initially creating the impeller pump platform, we selected low-cost materials that required no expertise or formal training to assemble with a build time of < 2 hrs. This was critical to make sure that the impeller pump would be accessible to researchers with limited microfluidics experience. The overall cost of materials for the pump (not including the chip or stir bar) was <$40, a significant decrease compared to $1000+ commercially available pumps. Due to the rapid fabrication and assembly times for the pump and low-cost materials, it is straightforward to build multiple pumps to run multiple chips simultaneously with an optional laser-cut acrylic pump holder for ease of use, especially when using the pumps in a cell culture incubator (Fig. 1d-e). We note that materials from vendors that provide more quality control may provide more reproducible pump performance at a higher up-front cost.

In terms of footprint and throughput, up to 72 of the current pumps can be fit, in principle, into a cell culture incubator. The pump footprint could be decreased more in the future by switching to a smaller DC motor and further compacting other components in the motor circuit such as the POT.

### 2.2 Design considerations for the 3D-printed impeller pump housing

**Fig. 2:**
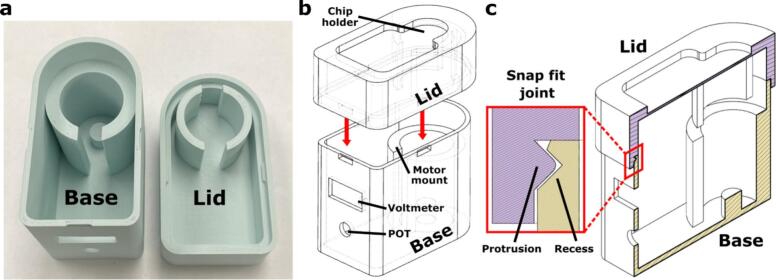
**Features of the Impeller Pump Housing.** (a) An image of the FDM 3D-printed Impeller Pump Base (left) and Impeller Pump Lid (right). (b) A schematic from Fusion 360 showing the assembly of the pump housing. The red arrows mark the snap fit joints. (c) A schematic from Fusion 360 of a central cut plane of the assembled pump housing. The snap fit joint consisted of a protruding feature on the lid (purple) and a corresponding recess on the base (yellow).

The Impeller Pump Housing is comprised of the Impeller Pump Base and Impeller Pump Lid, which were designed to fit the needs of our current OOC applications (Fig. 2a). For users considering other applications, we outline **critical** features that are required for proper pump function as well as features that are **customizable.**

The features that are **critical** include:

1) *Chip holder on lid of the pump housing (*Fig. 2*b).* This feature is built into the lid of the pump housing to position the device and the impeller over the DC motor. Consistent XY positioning of the impeller in the well is critical for consistent and predictable pump function. Currently, we position the impeller in the center of the well.
2) *Mount for DC motor (*Fig. 2*b).* The motor mount is included in the Impeller Pump Base to limit vibration or translation of the motor when using the pump at high voltages. It should be a tight fit, therefore the inner diameter of the motor mount may need to be adjusted depending on the FDM printer used and filament material.
  a. The small post in the center of the mount supports the base of the motor. An additional motor mount in the Lid aligns the lid and its chip holder over the DC motor.

The features that can be **customized** include:

1) *Openings for voltmeter, POT, wires, and other features in the motor circuit (*Fig. 2*b).* These openings or mounts for additional features added to the motor circuit can be easily added using any CAD software. An example of a feature that can be added is a power switch. The current design locates all of the holes in the pump base for simplicity when removing the lid.
2) *Snap fit connections (*Fig. 2*c).* These reversible connections were designed to allow for users to open the pump housing as needed to repair or replace parts of the motor circuit. More snap fit connections can be added as needed. Alternatively, for a permanent seal, you can remove the snap fit connections and use epoxy on the seam, as used in a prior version of the pump [15]. For a reversible seal that is more air-tight, you can adapt the connection to incorporate a gasket or O-ring.
3) *The number of motor circuits per pump*. Here, each pump housing contains a single motor circuit. To further condense the pump footprint and increase throughput, larger boxes may be designed to accommodate multiple motor circuits, provided that alignment features (motor mounts and chip holder) are maintained. We estimate that on a standard FDM printer with 150 x 150 mm^2^ build plate, pump housings for up to 6 motor circuits are feasible.

### 2.3 3D-printed microfluidic device compatible with impeller pump

The impeller pump is designed to drive flow through a companion microfluidic device. Here, we fabricated that device using resin 3D printing, which allowed for rapid fabrication of custom 3D geometries. A demonstration device is included with this publication; below we provide design considerations so that users may design a custom chip that suits their needs and is compatible with the impeller pump. As one example, we have added culture chambers along the loop of channels for tissue slice co-culture under recirculating fluid flow [15].

#### 2.3.1 Design considerations for the microfluidic device

**Fig. 3:**
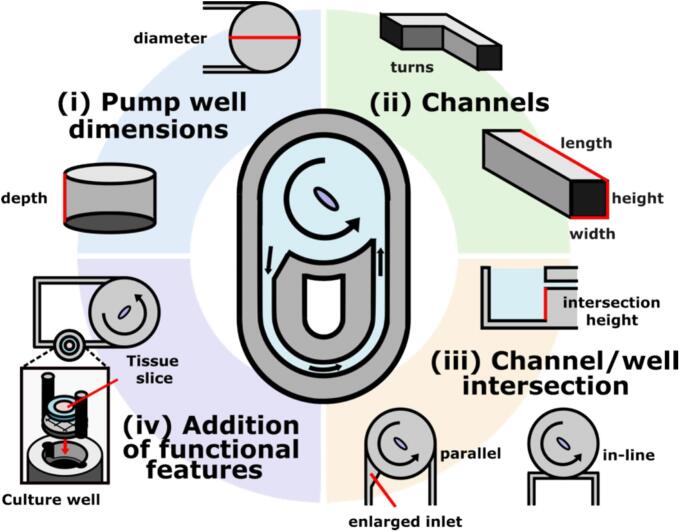
**Adjustable features of the 3D-printed device.** These features include (i) pump well dimensions, (ii) channel dimensions and corners, (iii) channel/well intersection, and (iv) addition of functional features (e.g. well for tissue slice culture).

Again, we outline **critical** features that are required to enable recirculating fluid flow on-chip and others that are **adjustable** to fit the needs of each use case (Fig. 3).

The features that are **critical** include:

1) A cylindrical pump well.
2) A loop of channels whose inlet and outlet are connected to the pump well.

The features that can be **adjusted** include:

1) Pump well dimensions (Fig. 3i).
  a. *Diameter.* An increase in diameter will reduce the channel speed. The current chip holder was optimized for a 15 mm pump well.
2) Channels (Fig. 3ii).
  a. *Width and height.* The channel speed increases with a larger channel cross-sectional area.
    i. *Note:* We have tested square channels between 0.25–1 mm^2^ using this pump.
  b. *Length of channels.* The channel speed decreases with longer channels as the resistance in the fluidic loop increases.
  c. *Turns*. Multiple turns adds resistance to the fluidic loop, resulting in reduced channel speed. In addition, sharp corners may impact the viability of recirculating cells.
3) Channel/well intersection (Fig. 3iii).
  a. *Intersection height and pump well depth.* Increasing the height at which the inlet and outlet intersect with the pump well moves the channels further from the rotating impeller, which reduces pressure from the fluidic vortex and decreases channel speed. When this is paired with increased pump well depth and diameter, further channel speed reduction can be achieved.
  b. *The inlet size and shape.* Traditionally, centrifugal pumps rely on a one-way valve at the outlet to prevent backflow and keep unidirectional flow. In place of a one-way valve, the current design has an enlarged inlet, where the wide part of the channel intersects with the pump well. The wider channel offers less resistance and greater cross-sectional area for entry, therefore encouraging the fluid flow consistently in the same direction. While this method has been useful, we do not know if this inlet geometry will work for every device design, so users may consider other methods, like a one-way check valve or Tesla valve.
  c. *Geometry of channel intersection with the pump well.* The orientation of the channels relative to the pump well can be manipulated to determine the direction of flow based on the direction of fluid momentum in the well at the point of intersection. For example, the current design has parallel channels entering on either side of the well, directing flow in the same direction as stir bar rotation [14]. One may alternatively position inlet and outlet channels in line along a tangent to the edge of the pump well to direct flow in the opposite direction of stir bar rotation [15]. Additionally, we have shown that reversing the direction of impeller rotation reverses the flow direction through the channel, as expected [14].
4) Addition of functional features such as culture wells or sensor openings (Fig. 3iv). This is dependent on the use of the device and what cell and tissue model(s) would be integrated into the device (e.g. a reservoir could be added in the fluidic loop for tissue slice culture).

#### 2.3.2 Potential limitations regarding generation of microplastics

In this version of the pump, the impeller rotates directly atop the surface of the 3D printed pump well. Although lubricated by the fluid in the well, we have observed that after overnight use, scratches appear in the bottom of the well. It is likely that this damage to the plastic surface will generate micro- and nanoparticles, which may circulate to reach any cells or organs cultured in the device. We have not assessed the presence or impact of microplastic generation, the extent of which likely varies with the viscosity of the fluid, the speed of impeller rotation, and the material used to make the chip. However, we have demonstrated preserved viability and function of lymph node tissue slices after 24 h when using this system [15].

### 2.4 Hardware summary

● A microscale impeller pump capable of recirculating flow through microfluidic and organ-on-chip devices.
● Translatable to labs with little microfluidics experience: no tubing required, low cost, and easy to use.
● Compatible with cell culture incubators.
● Achieves fluid velocities ranging from µm/s to mm/s.
● Customizable pump and 3D-printed chip geometry for a wide range of applications.
● Low-cost materials and fabrication

## 3 Design files summary

Design files for the 3D-printed microfluidic device (“demo chip”), the 3D printed components of the pump housing and motor assembly, and a laser cut pump holder are provided in Table 1 with descriptions of each below the table.

**Table 1:** Impeller pump and demo chip design files.

**Design file name**	**File type**	**Open-source license**
Demo chip	STL file	CC BY-NC 4.0 Files available at https://doi.org/10.17632/5njjj7kpys.1
Magnet Mount	STL file	
Impeller Pump Base	STL file	
Impeller Pump Lid	STL file	
Chip Cover	STL file	
Cross Impeller	STL file	
Pump Holder Top	DXF file	
Pump Holder Bottom	DXF file	

**Demo chip** is an STL file of the device used for demonstration within this paper. Dimensions included in Fig. S1.

**Magnet Mount** is an STL file of the component containing magnets that will be mounted on the motor shaft.

**Impeller Pump Base** is an STL file of the housing base for the impeller pump. Dimensions included in Fig. S2c,d,e.

**Impeller Pump Lid** is an STL file of the housing lid for the impeller pump. Dimensions included in Fig. S2a,b,e.

**Chip Cover** is an STL file of the chip cover for use within cell culture incubators. Dimensions included in Fig. S3.

**Cross Impeller** is an STL file of the cross-shaped impeller housing from prior work that we use here to measure pump rotational speed (Section 7.1) [14].

**Pump Holder Top** is a DXF file of the 2D vector outlines of the top layer of the laser-cut acrylic tray used to hold 6 pumps.

**Pump Holder Bottom** is a DXF file of the 2D vector outlines of the bottom layer of the laser-cut acrylic tray used to hold 6 pumps.

## 4 Bill of materials summary

*Power supply cable is used for 6 single-motor pumps.

### 4.1 Description of motor circuit components

**Fig. 4:**
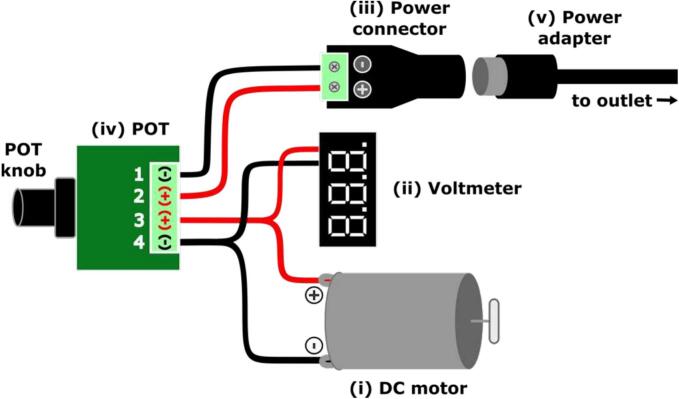
**Motor circuit diagram.** Schematic showing how wires from the (i) DC motor, (ii) voltmeter, and (iii) power connector are attached to the positive and negative terminals of the (iv) POT. A (v) power adapter is used to connect the (iii) power connector to an outlet.
ii. *Voltmeter (*Fig. 4*ii).* The voltmeter reports the voltage that the motor is receiving, allowing for user tunability.
iii. *Power connector (*Fig. 4*iii).* The power connector provides the motor circuit with power, and easily plugs in to the cable from the power adapter.
iv. *Potentiometer (POT) (*Fig. 4*iv).* The POT modulates the voltage that the DC motor receives, where higher voltages result in higher RPM. The POT that we selected functions by rotating a knob that is outside of the 3D-printed housing.
v. *Power adapter (*Fig. 4*v).* The power adapter we use plugs into two-prong US wall outlets. This adapter also comes with a cable splitter, which enables users to use a single power adapter for up to 8 different pumps.

i. *DC motor (*Fig. 4*i).* This component is used to rotate magnets within the pump housing below the device. The initial impeller pump design used computer fans due to their negligible heat output and low cost, but we have since switched to DC motors to reduce the pump size [14]. DC motors often generate a lot of heat, which would be an issue when using these pumps with biological samples within a cell culture incubator. However, we found that these motors performed acceptably at a low voltage: the temperature within an incubator remained within an acceptable range (+/- 1 °C) with 8 pumps operating at 4 V over a period of 10 days [15].

## 5 Build instructions

The additional tools required for fabrication and assembly included:

• FDM printer.
• Wire stripper.
• Soldering iron and solder material.
• Wire helping hands (optional).
• Heat gun or lighter.
• Hot glue and Super glue.
• Digital tachometer for RPM measurement.
• Digital light processing (DLP) printer.
• Laser cutter (optional, for Pump Holder).

### 5.1 Fabrication of pump components

#### 5.1.1 FDM 3D printing of pump housing and chip Cover

The Impeller Pump Lid, Impeller Pump Base, and Chip Cover were designed using Fusion 360 and printed with a Creality Ender-3 V3 KE 3D printer and Creality Print slicing software in 1.75 mm PLA filament listed in Table 2. It is expected that any FDM printer with a build size of at least 150 x 150 mm would be able to print the pump housing and Chip Cover, though print settings may need to be adjusted depending on the printer resolution.

1) For the Impeller Pump Base, orient the print with the bottom of the print on the build plate. For the Impeller Pump Lid, orient the print with the top of the lid on the build plate and use supports as these are required to print the chip holder component of the lid. For the Chip Cover, orient the print with the top of the cover on the build plate.
2) For each component, slice and print the files provided in Table 1 using the following settings:
  a. 210C nozzle temperature, 60C build plate temperature, 25 % infill density, 30 mm/s initial layer print speed, 100 mm/s support speed, 150–400 mm/s normal print speed, 0.5 mm initial layer line width, and 0.4–0.42 mm normal line width.
  b. Note: the parts are not sensitive to print settings and likely could be printed under a range of parameters.
3) For the Impeller Pump Lid, supports are required using the following settings:
  a. 15 % support density, triangle pattern, 0.2 mm support layer thickness.
4) Once printed, the Impeller Pump Base and Chip Cover require no post-processing. For the Impeller Pump Lid, gently detach the print supports.

**Table 2:** Materials for the impeller pump.

**Designator**	**Component**	**Units (per motor circuit)**	**Cost per unit − USD**	**Total cost − USD**	**Source of materials**	**Material type**
Demo chip	CADworks3D Clear Microfluidics Resin V7.0a	1 (3.70 mL)	$510.00/1 L	$1.89	CADworks3D	Polymer
5 mm stir bar	Micro stir bars, Teflon PTFE coated, 2x5 mm (Cat# 58948–377)	1	$9.28	$9.28	VWR	Metal/ polymer
10 mm stir bar	Teflon-encapsulated magnetic stirring bar, 3x10 mm (Cat# 8608S92)	1	$6.28	$6.28	Thomas Scientific	Metal/ polymer
DC motor	6–12 V mini high torque DC motor	1	$6.89	$6.89	Amazon AutoToolHome Store	Metal
Potentiometer and knob	PWM low voltage DC motor speed controller, 3 pcs	1	$3.00	$3.00	Amazon ALEDECO Store	Other
Voltmeter	Mini digital DC voltmeter, 2.5–30 V, 5 pcs	1	$1.92	$1.92	Amazon MakerFocus	Other
Disk magnets	6 x 2 mm brushed nickel magnets, 300 pcs	2	$0.03	$0.06	Amazon DIYMAG Store	Metal
Magnet Mount	CADworks3D Clear Microfluidics Resin V7.0a	1 (0.57 mL)	$510.00/1 L	$0.29	CADworks3D	Polymer
Power connector	Female 12 V DC power connector, 10 pcs	1	$0.90	$0.90	Amazon CHANZON Store	Other
Power adapter	12 V 5A AC adapter, 1 x 1–8 splitter cable	1*	$14.60	$14.60	Amazon ARyee Store	Other
FDM filament for Impeller Pump Lid	1.75 mm Matte PLA filament, 1 kg spool, seafoam blue	1 (36 g)	$25.99/kg	$0.94	Amazon HATCHBOX Store	Polymer
FDM filament for Impeller Pump Base	1.75 mm Matte PLA filament, 1 kg spool, seafoam blue	1 (54 g)	$25.99/kg	$1.40	Amazon HATCHBOX Store	Polymer
FDM filament for Chip Cover	1.75 mm Matte PLA filament, 1 kg spool, seafoam blue	1 (12 g)	$25.99/kg	$0.31	Amazon HATCHBOX Store	Polymer
Wire	22-gauge stranded tinned copper wire, 200 ft black and 200 ft red	∼170 cm	$28.99/400 ft	$0.40	Amazon OPLIAT Store	Metal/ polymer
Heat c	Wire wrap sleeve, 3 mm diameter, 30 mm long, 240 pcs in kit	1	$6.49/kit	$0.03	Amazon uxcell	Polymer
Acrylic for Pump Holder	Clear acrylic sheet, 1/4″ thick, 12 x 12 in, 2 sheets	1	$14.99/2 sheets	$7.50	Amazon CALPALMY Store	Polymer
Cross Impeller [14]	CADworks3D Clear Microfluidics Resin V7.0a	1 (0.57 mL)	$510.00/1 L	$0.26	CADworks3D	Polymer
Food Coloring	McCormick Culinary Blue Food Coloring	1 (16fl oz)	$11.99/bottle	$11.99	Amazon McCormick Culinary Store	Other

#### 5.1.2 Laser cutting of optional holder for multiple pumps

An OMTech 70 W CO_2_ Laser Engraving/Cutting Machine and Lightburn software were used to cut the Pump Holder Top and the Pump Holder Bottom. The corresponding files and materials are listed in Table 1, Table 2, respectively.

1) To make both layers of the Pump Holder, laser cut a 1/4 in thick sheet of acrylic using the files provided for the pump holder using the following settings:
  a. 100 % power, 6.0 mm/s speed, 2 pass count.
  b. Make sure the cut is “Line” and not “Fill” or “Raster.”
  c. Note: settings will likely vary depending on the laser cutter used.
2) To assemble the pump holder, the two layers were glued together using super glue.

#### 5.1.3 DLP 3D printing of the demo chip and Magnet Mount

The Demo Chip and Magnet Mount were designed using Fusion 360 and printed using a CADworks3D MiiCraft Prime Series Pr110-385 nm resin DLP printer. Each part was printed in commercially available CADworks3D Clear Microfluidics Resin V7.0a. We have successfully used other resins as well, including MiiCraft Clear and house-made PEGDA resins [18,19]. The microfluidic device included here was designed for this demonstration only and may serve as a starting point for customization. The minimum internal channel size able to be printed is determined by the resolution of the 3D printer, the settings used, and the composition of the resin. Using this printer and resin, we have printed internal square channels as small as 0.04 mm^2^ [20].

1) For each print, the design was sliced at 50 µm thickness and printed using the settings below. The print was oriented such that the bottom of the device was against the build plate.
  a. 0.85 S cure time, 3.00 s base cure time, 3 base layers, 2 buffer layer, and 75 % power (3.75 mW/cm^2^).
2) Once complete, the print was removed from the build plate and cleaned in an isopropanol-filled Form Wash (Formlabs) for 4 min. The channels were flushed with additional isopropanol to remove any leftover uncured resin.
3) The prints were then dried thoroughly with compressed air and placed in the Form Cure UV light box (10 mW/cm^2^; Formlabs) for 2 min at room temperature.
  a. Note: the post-processing procedure for devices may vary if using other resins or if using the 3D-printed chip with biological samples, e.g. increased cure time and temperature. For biocompatibility with primary cells, we coat the 3D printed devices with Parylene C [20,21].

### 5.2 Assembly of impeller pump

The motor circuit is comprised of a (i) DC motor, (ii) POT, (iii) voltmeter, (iv) power connector, and (v) power adaptor (Section 5.3.1). These components are assembled with minimal soldering and a screwdriver.

**Fig. 5:**
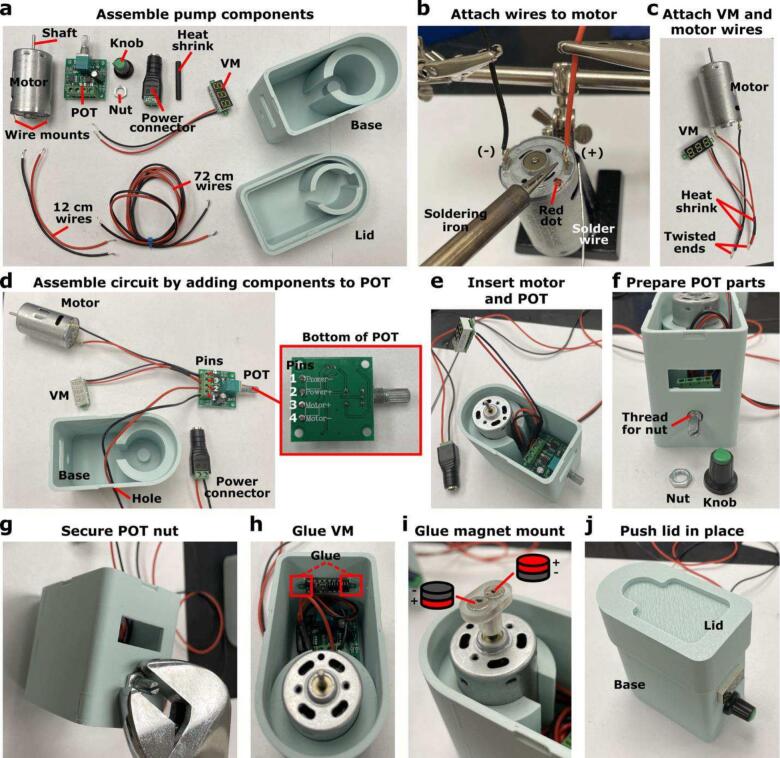
**Assembly of impeller pump external housing.** (a) Components required for pump assembly, (b) wires attached to the DC motor, (c) heat shrink used to hold twisted voltmeter and motor wires together, (d) wires inserted into the POT and power connector, (e) the motor and POT secured in the Impeller Pump Base, (f) components to secure the POT in place, (g) POT nut tightened with a wrench, (h) voltmeter glued into place, (i) disk magnets glued to the Magnet on the motor shaft, and (j) Impeller Pump Lid snapped into place to finish pump assembly.
2) Cut two ∼ 12 cm wires and two ∼ 72 cm wires, one red and one black for each length. Strip ∼ 0.5 cm of insulation off each end of all wires using the wire stripper.
3) To attach wires to the DC motor, loop one end of the red 12 cm wire through the wire mount of the DC motor that is closest to the red dot. Repeat with the black 12 cm wire on the opposite wire mount.
4) Solder the looped wires to the respective wire mounts (Fig. 5b). We recommend soldering both the front and the back of the wire mount to have a stable connection. Tug gently on the wires to ensure they are secured.
5) To connect the wires of the DC motor and voltmeter, twist together the exposed ends of the red wires connected to the DC motor and the voltmeter. Cut the heat shrink in half (optional), and thread one half of the heat shrink on the twisted red wires. Use a lighter or heat gun to tighten the heat shrink on the wires, holding both wires together. Repeat with the black wires (Fig. 5c).
6) Insert the red and black wires from the DC motor and voltmeter into pins 3 (+) and 4 (−), respectively, on the POT (Fig. 5d). Use the screwdriver to tighten the wires in the pins. Tug gently on the wires to ensure they are secured.
  a. Note: limit the amount of exposed wires that stick out from the POT pins. There is the potential for the wires in different pins to touch, and if the exposed portions touch, it will cause pump failure.
7) Connect the red and black 72 cm wires to the power connector by inserting the exposed ends into the (+) and (−) pins, respectively, screwing the wires in place. Tug gently on the wires to make sure they are secured. Loop the other end of the 72 cm wires through wire hole in pump housing and insert the black and red wires into pins 1 (−) and 2 (+), respectively, on the POT (Fig. 5d). At this stage, the entire motor circuit is assembled.
  a. Note: it is useful to conduct a quality control test on the motor circuit at this point (see Section 5.3).
8) Insert the motor into the motor mount in the Impeller Pump Base (Fig. 5e). The wire mounts should be facing down and the motor shaft should be facing up. The wires connected to the wire mounts should be coming out of the gap in the front of the motor mount.
9) Insert the POT into the Impeller Pump Base and place the metal POT knob through the hole on the front of the Impeller pump base (Fig. 5e). Hold the POT in place inside the Impeller Pump Base and screw on the nut outside of the Impeller Pump Base using a wrench (Fig. 5f-g). The nut should be tight enough where the metal POT knob can be rotated without moving the POT inside the Impeller Pump Base. Place the plastic knob on the metal knob with the point facing down (optional).
10) Ensuring the voltmeter is facing outward, use hot glue to secure the voltmeter in the appropriate hole in the Impeller Pump Base (Fig. 5h).
11) Glue two disk magnets of opposite polarity in the Magnet mount using superglue (Fig. 5i). Slide the Magnet Mount on to the motor shaft, making sure that the top of the shaft is touching the top of the hole in the mount.
  a. Note: the distance between the rotating magnets and rotating impeller were optimized to ensure stable rotation. It is important to ensure that there is no gap between the tip of the motor shaft and the Magnet Mount, both to maintain the proper distance between the magnets and impeller and to make sure the rotating Magnet Mount does not hit the pump lid.
12) Finally, align and push down the Impeller Pump Lid on top of the fully assembled Impeller Pump Base to snap in place (Fig. 5j, Movie S1). The snap fit joint will make a sound when it has properly fell into place (*see* Movie 1 *in supplementary files for demonstration*).
  a. Note: the snap fit joint was designed to be impermanent. To remove the Impeller Pump Lid, grip either side of the lid near where the snap fit joints are. Pull out slightly, and lift the lid off, pulling directly upwards without tilting the lid. The lid should also come off if you pull hard enough, and will likely wear down faster if you use that removal method.
  b. Note: make sure to do the quality control test here if not in Step 8 (see Section 5.3.3).

1) Gather required pump components (Fig. 5a).
  a. DC motor.
  b. POT.
  c. Nut for POT attachment.
  d. POT knob.
  e. Voltmeter.
  f. Heat shrink tubing.
  g. Red and black wire.
  h. Impeller Pump Base.
  i. Impeller Pump Lid.
  j. Disk magnets (x2).
  k. Magnet Mount.

### 5.3 Impeller pump quality control and troubleshooting

Due to the low-cost materials used, there can be variance in the performance of the components of the pump. It is important to do a simple quality control test with each pump during or after assembly to guarantee that it is functioning properly:

1) Remove the Impeller Pump Lid. Attach a small piece of reflective tape, approximately 2 x 2 mm, on one of the magnets on the Magnet Mount.
2) Make sure the POT is fully turned off and plug the pump into a wall outlet.
  a. Note: if the POT is on when plugged in, higher voltages may result in the motor moving significantly if not inserted into the pump housing, or a loud, alarming sound.
3) Slowly turn the POT knob to power the motor circuit. The motor shaft should begin rotating at approximately 1.2–1.5 V.
  a. Note: the motor may require a higher voltage to initially start rotating but then can be lowered to 1.2–1.5 V.
4) Measure the RPM of the Magnet Mount at the lowest voltage with rotation (∼1.2 V) using a digital tachometer (KAISAL). Repeat 3–4 times.

#### 5.3.1 Quality control checklist

• Does the voltmeter light up and display a voltage when turned on? It should show a range of voltages between 0.01–12 V.
• At a constant POT knob position, how much does the voltage fluctuate? Fluctuations should be within +/- 0.05 V.
• Does the motor shaft rotate at voltages greater than 1.2–1.5 V? It should start rotating above this voltage, and the rotational speed should appear to increase as the voltage increases.
• Does the RPM measured at low voltage (step 4) fall within the acceptable range of 800–1000 RPM? The RPM for each motor circuit should be relatively similar; if one circuit is significantly slower, see troubleshooting below.

If any of the items on the checklist were not met, parts of the motor circuit may need to be replaced.

Caution: When working with the motor circuitry, always unplug the power adapter to avoid electrical injury.

#### 5.3.2 Troubleshooting

1) *High fluctuation of voltages shown on voltmeter*.
  a. We have noticed this issue with pumps that have been used long-term (6–12 months) or with individual voltmeters that may be old or have been damaged.
  b. This problem has been resolved by replacing the voltmeter.
2) *The voltmeter starts at a higher voltage than expected*.
  a. We have noticed this issue when connecting a newly assembled motor circuit to power for the first time.
  b. We suspect this issue is specific to the voltmeter, therefore it can be resolved by replacing the part. If the issue persists, replace the parts in the following order: POT, motor, and power connector.
3) *The motor RPMs are much lower than expected for a specific motor circuit*.
  a. We suspect this issue is related to variation with each DC motor, as the product is inexpensive and likely not intended for high accuracy.
  b. To solve this issue, replace the motor in the circuit with a new motor. A long-term solution to this issue is to use more accurate and consistent motors from a different source, though this will increase the overall cost of the pump platform.
4) *The pump is not receiving power*.
  a. There are a number of reasons why this issue can occur. We have primarily seen this issue with pumps of a previous design that were used long-term (6–12 months) in an incubator.
  b. To solve this issue, try the following steps in order, stopping once the issue is solved.
    i. Check wire connections throughout entire motor circuit and fix/replace if there is an unconnected, loose, or damaged wire. In addition, ensure the stripped parts of the wires are only as long as needed to fit inside any electronic component. If the stripped wire is too long, it may encounter another stripped wire resulting in a short circuit.
    ii. Test power adapter with a pump known to work to confirm that the power adapter has not been damaged.
    iii. Replace the power connector with a new part.
    iv. Replace the POT with a new part.
    v. Replace the voltmeter with a new part.

## 6 Operation instructions

### 6.1 Filling the 3D-printed device (Movie S2)

The procedure for filling these 3D-printed devices may vary depending on the features present within the device, e.g. tissue culture wells. Included below are areas of potential issue with tissue culture wells added.

1) Using your solution of choice, add 800 µL into the pump well using a pipette. In this case, we used phosphate-buffered saline (PBS).
  a. Note: this volume was determined to be sufficient to fill the channels and the pump well while the stir bar rotated. With different designs, the fill volume may vary.
2) To fill the channels, collect ∼ 70–90 µL from the pump well and inject through the ports of the channels. Ensure there are no air bubbles on the tip of the pipette before filling the channels at any point to limit bubbles. Repeat for each port present.
  a. Note: for the demo chip shown in Fig. 1b, there are only two ports, but additional ports would be required for chips with longer channels or channels with frequent turns.
3) When dry, the channels appear to be white. When properly filled, they will be the color of the solution, e.g. clear when using PBS. If present in the channels, bubbles will be visible as white spots. To remove the bubbles, take ∼ 70–90 µL of liquid from the pump well and gently pulse in and out of the port. This pulsing movement can help move the bubbles to a nearby port to be removed.
  a. Note: if pulsing does not remove the bubble, more suction may be required. Try pipetting out of the port only.
4) Once the channels are filled and bubbles are fully removed, confirm chip filling by pulsing solution from the pump well in either port. If the channels are filled with no bubbles, movement of the liquid in other port(s) is visible upon pulsing.

### 6.2 Operation of the motor-based impeller pump (Movie S3)

1) Place the chip on top of the pump housing with the pump well resting inside the chip holder.
2) Place a stir bar in the pump well.
  a. Note: if using a 3D-printed impeller, make sure it aligns with the magnets in the Magnet Mount and make sure the impeller did not flip over upon insertion.
3) Make sure the POT is fully turned off and plug the power adapter into a wall outlet. Set the voltage by turning the POT knob clockwise.
  a. Optional: confirm that fluid is recirculating on-chip and in a consistent direction by pipetting a small droplet of food coloring to either a channel port or the pump well.
  b. Note: if using a 3D-printed impeller, ramp up the voltage slowly when turning on the pump as it can be more sensitive and may misalign with the rotating magnets below.

### 6.3 Operation instructions for use in a cell-culture incubator

When using the motor-based impeller pumps within a cell culture incubator, place the pump(s) directly within the incubator on a shelf. The power connector that is plugged into the power adapter should remain outside the incubator to limit humidity damage. All wires and power cables must be threaded under the rubber gasket of the incubator door or may be fed through the port in the back of the incubator if available. We recommend covering any device with a Chip Cover before use in an incubator to limit evaporation.

### 6.4 Pump operation troubleshooting

1) *The stir bar is not rotating smoothly.* This is common when the magnets are rotating too fast. As for most magnetic stir bars, there is an upper limit to how fast the stir bar can rotate within the pump well before it becomes unstable. For the motor-based pumps used here, that limit is around 3.5–4 V. To solve this issue, use the impeller pump at a lower voltage. Alternatively, if you slowly ramp up the voltage, that may help the stir bar stability.
  a. If you need a faster stir bar rotation to achieve a faster speed, consider altering the dimensions of the device (e.g. channel size, pump well diameter, etc.) and impeller shape and size to increase the channel speed while still maintaining a stable voltage. See section 5.1.3 for more details.
2) *Inconsistent dye movement, such as switching fluid flow direction, jumping forward quickly, or no recirculation*. Partial or total channel blockage by air bubbles impacts the pump performance. If bubbles are present, remove as described above.
  a. If the stir bar or impeller is misaligned within the pump well, this can cause fluctuations in flow direction. We have optimized the pump housing to attempt to have consistently centered stir bars and impellers between pumps, however realignment may be necessary.
  b. If the flow direction continues to be inconsistent, we found that enlarging the channel inlet helps drive flow consistently one way (Section 2.3.1). The addition of this feature can result in an increase in channel speed.
  c. If the fluid is jumping forward quickly, switching flow direction, or not recirculating at all, the flow resistance within the channel may be too high. You can overcome the resistance issue by either increasing the impeller size, pump voltage, or alter the device geometry (Section 5.1.2).

## 7 Validation and characterization

### 7.1 Characterization of impeller function

#### 7.1.1 Impeller RPM as a function of voltage on the pump

We measure impeller RPM as a function of voltage for each pump as a final quality control before further use, and to link all pump-related experiments to the RPM rather than the pump voltage. RPM can be measured as described in 5.3. Alternatively, if measured on-chip, the reflective tape could be added to one side of an impeller. When using a stir bar as an impeller, users may have difficulty adding the reflective tape to the Teflon-encapsulated material, so we quantify pump RPM with a 3D-printed impeller that the stir bar is loaded into, as described previously [14].

**Fig. 6:**
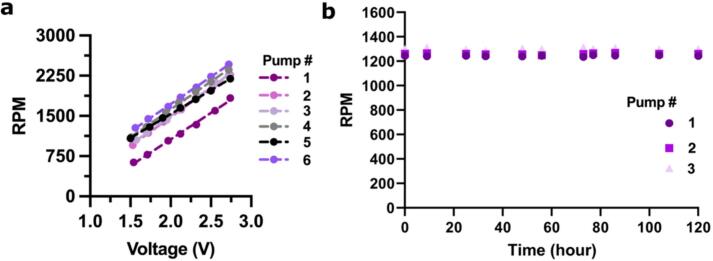
**Characterization and stability of impeller.** (a) As the voltage increased, the impeller RPMs increased for pumps 1–6. Note pump #1 was slower than the others and failed the quality control test. (b) RPM of the impeller remained stable across 3 pumps for 5 days at 1.7 V.

The RPMs of each motor vary slightly, even at the same voltages. To illustrate this, we measured the impeller RPMs at a range of pump voltages for six pumps. As expected, RPMs increased with voltage (Fig. 6a) [14,15]. However, pump 1 had a significantly lower range of RPMs compared to the other five pumps (Fig. 6a), indicating a defective DC motor that must be replaced. We try to catch this issue at earlier stages in pump assembly using the quality control tests described in Section 5.3.

#### 7.1.2 Impeller stability over extended use

To reliably perform experiments over an extended period of time, it is crucial that the rotation speed of the impeller remains constant over time. To confirm this, we measured the RPM of the impeller in 3 pumps at the same voltage (1.7 V) over a 5-day period (Fig. 6b). Chips were filled and ran per 6.1, 6.2. The RPMs were measured by adding a small piece of reflective tape to one end of the upward facing side of each stir bar and using a digital tachometer (KAISAL). The rotation speed remained stable over this time for each pump with an average coefficient of variation of 0.48 % (SD = 0.17).

### 7.2 Characterization of channel velocity at different pump speeds

The impeller pump can achieve velocities on the order of µm/s to mm/s within the channel [14,15], which increase linearly with pump voltage and decreases with resistance in the microfluidic channel. At these velocities, convective transport dominates over diffusive transport. Resistance varies with channel geometry and due to inclusion of extra modules, e.g. an organ-on-chip module, so velocity must be characterized as a function of RPM for each individual design of the microfluidic device. We found that it was challenging to measure the channel velocity using an in-line flow meter due to the high resistance of the meter. We developed an alternative method to track the movement of dye in the channel and extrapolate the maximum channel velocity [14]. This method involves two steps: (1) Tracking of dye using video and (2) video analysis using ImageJ.

#### 7.2.1 Tracking of dye using video

**Fig. 7:**
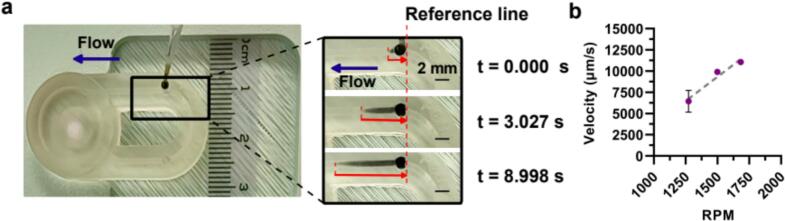
**Experimental maximum channel velocity quantification using the microscale impeller pump.** (a) Time-lapse images of dye movement in the channel of the demo chip (1270 RPM, 1.8 V) at different time points (0.000 s, 3.027 s, 8.998 s). The blue arrow notes the flow direction. The solid red line (arrow) indicates the distance from the tip of the parabola to the reference line, used to quantify the dye movement. The red dashed line marks the reference line used to measure dye movement over time, demonstrating the velocity measurements. (b) Experimentally measured maximum channel velocity within the demo chip as a function of RPM, showing an increase with higher RPM values. Dots and error bars represent mean and standard deviation (n = 4); some error bars are too small to see.
3) Place an iPhone 12 or similar cell phone or camera on a mini tripod stand for stable video capture. Ensure a ruler is placed next to the chip for scale and within the camera’s frame. We used the Timestamp Camera Basic application for video recording.
  a. Note: To increase accuracy, make sure the timestamp reports the time in seconds with up to 3 decimal places.
4) Begin recording the video, then add 1 µL of food coloring into a port of choice.
  a. Note 1 *Choose a port that is followed by a straight channel of at least* 2 *cm in length, to facilitate imaging in a straight segment.*
  b. Note 2: *It is best to wait until the flow re-stabilizes after injections to begin your measurement, about* 2–3 s*.*
  c. Note 3 *We used blue food coloring for all velocity measurements because it is the easiest to visualize within the device compared to red or yellow dye.*
  d. Note 4: *If the flow direction deviates from the expected direction, there may be a bubble present in the channel providing resistance, see* 6*.*1 *step* 4 *for removing bubbles.*
5) Once the dye has moved at least 2 cm down the channel, stop the video.
6) To clear the dye from the channels, pipette out 100 µL slowly from the port to remove most of the dye that was added. Replace the volume removed with fresh PBS, maintaining a constant fill volume within the device, and flush the channels to clear any remaining dye out of the region where velocity is measured.
  a. Note: if any bubbles are introduced when removing dye or flushing the channels, pulse the pipette (Section 6.1) to remove bubbles before beginning the next velocity measurement.
7) Repeat the dye insertion at least four times at a given voltage or other pump condition.
8) After changing the pump voltage or other pump condition, wait 3–5 min for the fluid flow to stabilize. Repeat steps 3–5 as needed.

1) Fill the device as described previously (Section 6.1, Movie S2). Load the device onto the pump and insert the stir bar or impeller of choice. Set the pump to the desired voltage. Allow an equilibration period of approximately 3–5 min to achieve a consistent velocity after initially starting the impeller rotation.
2) Add a line to the device as a reference point for measuring the distance dye moves over time (Fig. 7a).

#### 7.2.2 Video analysis using ImageJ

1) Collect at least 4 screenshots from the video that show the dye at different locations within the channels, no more than about 2 cm from the insertion port to minimize the impact of diffusive broadening.
2) Import the screenshots in ImageJ for analysis. ImageJ is an open-source software available for download at https://imagej.net/ij/download.html.
3) Set the scale using the ruler included in the video, converting pixels to µm.
4) Select a reference point that will be consistent for all images, either the edge of the port (Fig. 7a) or a line drawn on the device before recording the video.
5) For each image, record the time (s) listed on the image timestamp. Draw a line in the center of the channel, from the tip of the dye to the reference line. Measure the length of the line.
  a. Note: As expected for laminar flow, a parabolic flow profile is present with the impeller pump [14]. By measuring the tip of the parabola over time, we are measuring the maximum channel velocity.
6) Plot the distance travelled over time. The slope of the trendline is the maximum channel velocity in µm/s.
7) Average the replicates at a single RPM and plot the velocity as a function of RPM (Fig. 7b).

For this experiment, we measured the maximum channel velocity within the demo chip, specific to its geometry and stir bar. The results demonstrated a clear correlation between RPM and velocity, as expected.

### 7.3 Demonstration of recirculation of dye using different stir bar sizes

**Fig. 8:**
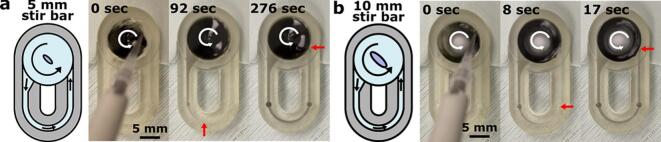
**Fluid recirculation using the impeller pump.** Schematic depicting flow direction and stir bar size alongside still images from video s of recirculating fluid flow using in the demo chip. Using a (a) 5 mm and (b) 10 mm stir bar at 1.7 V (1255 RPM), purple dye was added to the pump well and moved through the channel over time. The dye front is marked with a red arrow, and the stir bar rotation direction is noted with a white arrow. Scale bar = 5 mm. Stills are taken from Movie 4 and Movie 5 included in supplementary files.

We designed the impeller pump with the goal of recirculating fluid through a loop of channels at velocities ranging from µm/s to mm/s. Here, we demonstrated that fluid fully recirculated through the entire path of the looped channels at variable speeds by tracking the movement of food dye. The demo chip was filled with PBS (Section 6.1) and placed on the pump, which was then set to 1.7 V (1255 RPM). We recorded dye recirculation similar to velocity characterization (Section 7.2) by mounting an iPhone 14 parallel to the chip on a small tripod and recording a video using the Timestamp Camera app in order to monitor the time. After the pump equilibrated for 3–5 min, purple dye was added to the pump well at 0 sec and traveled counterclockwise through the channels (Fig. 8).

When using a 5 mm stir bar, the dye passed through the entirety of the channels and re-entered the pump well in approximately 276 sec (Fig. 8a, Movie S4). When a larger 10 mm stir bar was used, the dye traveled much faster as expected, completing the loop within 17 s (Fig. 8b, Movie S5). As demonstrated here, different flow regimes may be accessed by changing the stir bar size in addition to setting the pump voltage. Flow speeds may be further tuned based on the design features of the chip used, such as widening the channels (see Sections 5.1.2). Fine manipulation of each of these conditions can be used to achieved specific flow regimes for a wide range of microfluidic applications.

*Impeller pump capabilities* (Table 3):

• Stir bar RPM: 1050 to 2300 RPM at 1.5 to 2.7 V, respectively.
• Stable stir bar rotation from 1.5-2.7 V.
  o Limitation: unstable stir bar rotation at high voltages, 3–>4 V.
• Minor variability between stir bar RPMs at a given voltage for each pump.
• Fast load time for each chip: filling each chip should take < 2 min, and setting the pump to the ideal voltage should take < 1 min.
• Wide range of fluid velocities dependent on chip geometry, ranging from µm/s to mm/s channel speeds [14,15].
• Fluid recirculates through a microfluidic channel loop connected to the pump well.
• Minimal heat release with extended use of the impeller pump within a cell culture incubator [15].

**Table 3:** Comparison of impeller pump to traditional commercially available pumps.

	**Flow profile**	**Stable flow velocities (in 0.25 mm^2^ channel)**	**Cost and Accessibility**	**Setup considerations**
**Peristaltic pump**	Unidirectional; recirculation possible	mm/s to m/s	$100–1000 s	Extensive tubing, prone to leaking
**Syringe pump**	Unidirectional	µm/s to mm/s	$100–1000 s	Syringe loading and calibration
**Tubing-free impeller pump**	Unidirectional and recirculating	µm/s to mm/s	<$100, requires 3D printing	No external tubing, minimal pipetting

**Ethics statements**

SRC and RRP are listed as inventors on two patent applications (Serial No. 63/080320 and 63/543893) filed by the University of Virginia related to the impeller pump technology and multi-tissue culture.

## CRediT authorship contribution statement

**Sophie R. Cook:** Writing – review & editing. **Erin E. Lawrence:** Writing – review & editing, Writing – original draft, Validation, Formal analysis, Investigation, Methodology. **Parastoo Sakinejad:** Writing – review & editing, Writing – original draft, Validation, Formal analysis. **Rebecca R. Pompano:** Writing – review & editing, Supervision, Resources, Funding acquisition.

## Declaration of competing interest

The authors declare that they have no known competing financial interests or personal relationships that could have appeared to influence the work reported in this paper.
