# Supplementary material for: Open-source tubing-free impeller pump platform for controlled recirculating fluid flow for microfluidics and organs-on-chip
Source: HardwareX. 2025 Jul 4;23:e00673. doi: 10.1016/j.ohx.2025.e00673 (PMC12274319; doi:10.1016/j.ohx.2025.e00673)
Supplement: Supplementary Data 9 [file mmc9.docx]

Electronic Supplementary Information for:

**Tubing-free microscale impeller pump platform for controlled recirculating fluid flow for microfluidics and organs-on-chip**

Sophie R. Cook,^1^ Erin E. Lawrence,^1^ Parastoo Sakinejad,^2^ Rebecca R. Pompano^1,3^*

^1^Department of Chemistry, University of Virginia (UVA), Virginia, USA

^2^Department of Chemical Engineering, University of Virginia (UVA), Virginia, USA

^3^Department of Biomedical Engineering, University of Virginia (UVA), Virginia, USA

**Contents:**

- Supplemental Movie Captions
- Supplemental Figures

1. **SUPPLEMENTAL MOVIE CAPTIONS**

**Movie S1. Snap-fit lid attachment.** A video of the final pump assembly step, where the user presses on the lid of the pump box for the snap fit joint attachment.

**Movie S2. Filling the chip.** A video of filling the chip with liquid and clearing bubbles from the channels.

**Movie S3. Starting the pump.** A video of loading the chip on to the pump and turning the pump on using the potentiometer knob.

**Movie S4. Dye recirculation with 5 mm stir bar.** A video of dye being added to the pump well of the chip and moving through the channels using a 5 mm stir bar.

**Movie S5. Dye recirculation with 10 mm stir bar.** A video of dye being added to the pump well of the chip and moving through the channels using a 10 mm stir bar.

1. **SUPPLEMENTAL FIGURES**

**
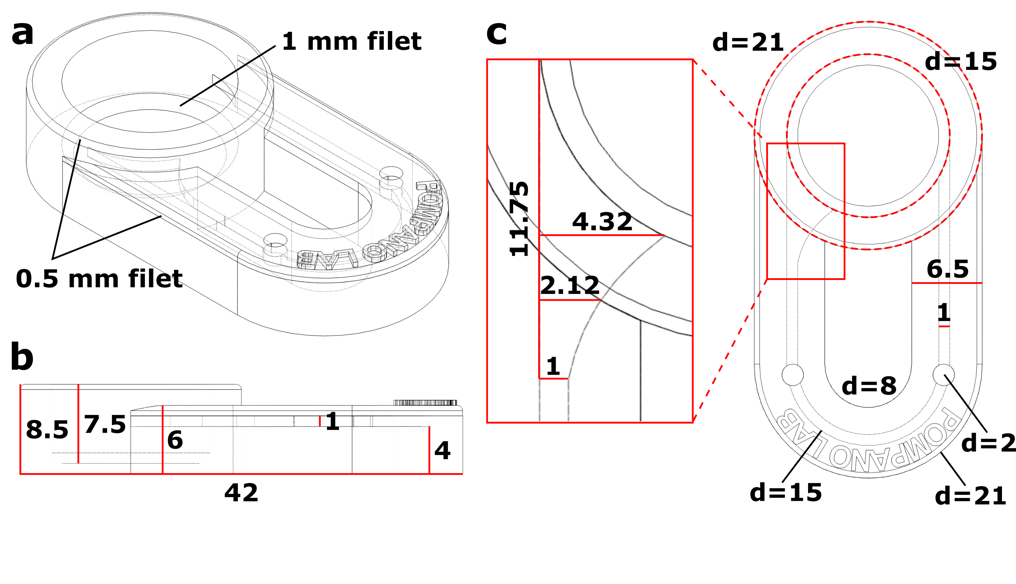
**

**Figure S1. Dimensions of demo chip.** A schematic from Fusion 360 with the dimensions of the demo chip from the (a) angled, (b) side, and (c) top view. Projection shows dimensions of enlarged inlet. All dimension in mm.

**
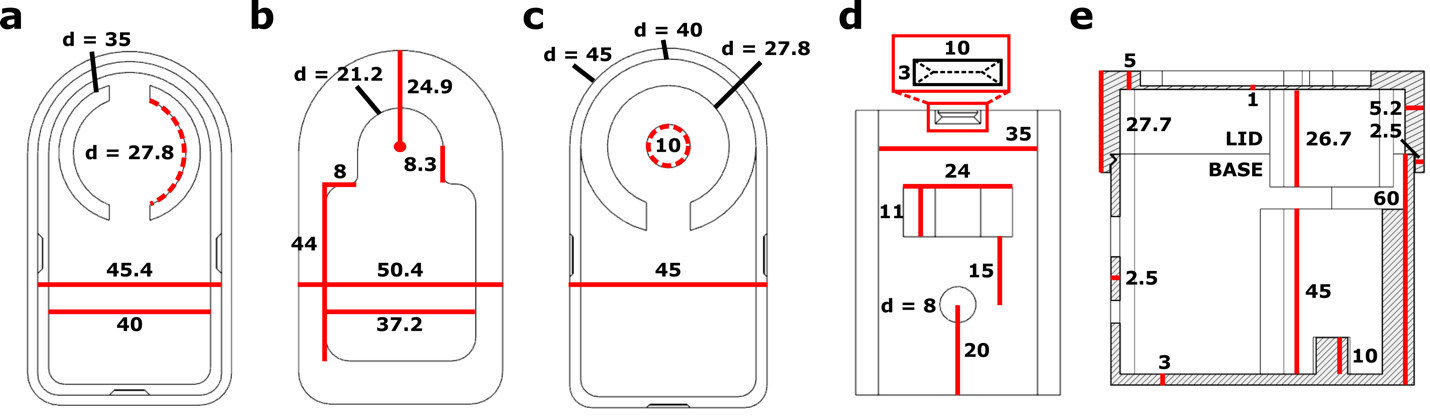
**

**Figure S2. Dimensions of impeller pump housing base and lid.** A schematic from Fusion 360 with the dimensions of the (a) bottom of lid, (b) top of lid, (c) top of base, (d) front of base, and (e) central cut plane of assembled pump box. All dimensions in mm.


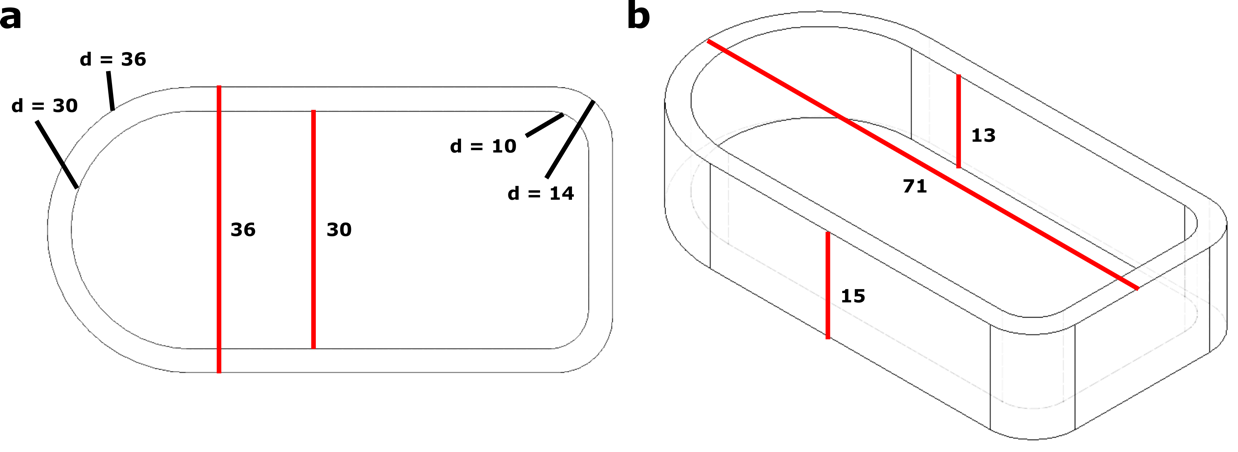


**Figure S3. Dimensions of chip cover.** A schematic from Fusion 360 with the dimensions of the chip cover from the (a) top and (b) angled view. All dimensions in mm.
